# A New Lens on Physical Activity Promotion: Can Technology Boost Exercise Prescription and Adherence?

**DOI:** 10.1093/geroni/igaa057.3089

**Published:** 2020-12-16

**Authors:** Patricia Heyn, Amber Watts

## Abstract

Adherence to exercise prescription for older adults (OAs) is a significant problem and can have a detrimental effect on key health outcomes. Exercise adherence for OAs is a multifactorial problem encompassing many factors affecting adherence such as socioeconomic status, education, physical fitness, and mental and health status. Improving exercise adherence could have a significant impact on longevity, quality of life, and health care costs. This symposium brings multiple perspectives to closely examine promising technology approaches, both in the form of models and programs. We will also discuss gaps regarding adherence to physical activity (PA) and exercise prescription for OA and the application of current publicly available technologies to boost PA adherence and compliance accordingly to the U.S. Department of Health and Human Services national standards for promoting health and preventing disease. The symposium includes five novel presentations addressing several key factors related to successful implementation of technology approaches to exercise program delivery and adherence for OAs. In addition, we will have one presentation highlighting the key factors that impact exercise prescription, compliance, and adherence. The speakers will present and address important components related to technology use with the goal to increase older adult’s PA participation. The exercise programs will target key areas affecting older adult’s health such as cognitive function, falls, obesity, gender, environments, and self-efficacy. Technology user-usability perspective will be presented. Current challenges and recommendations for future research will be comprehensively discussed to properly address the exercise adherence and compliance needs of our OA populations.

